# Cardiovascular Hypertension-Mediated Organ Damage in Hypertensive Urgencies and Hypertensive Outpatients

**DOI:** 10.3389/fcvm.2022.889554

**Published:** 2022-05-16

**Authors:** Fabrizio Vallelonga, Marco Cesareo, Leonardo Menon, Lorenzo Airale, Dario Leone, Anna Astarita, Giulia Mingrone, Maria Tizzani, Enrico Lupia, Franco Veglio, Alberto Milan

**Affiliations:** ^1^Hypertension Unit, Division of Internal Medicine, Department of Medical Sciences, Città della Salute e della Scienza Hospital, University of Turin, Turin, Italy; ^2^Division of Emergency Medicine, Department of Medical Sciences, Città della Salute e della Scienza Hospital, University of Turin, Turin, Italy

**Keywords:** hypertensive urgencies, left ventricular hypertrophy, arterial stiffness, blood pressure control, hypertension organ damage

## Abstract

**Background:**

The prevalence of hypertension mediated organ damage (HMOD) in patients attending the Emergency Department (ED) with symptomatic blood pressure (BP) rise is unknown, and whether HMOD varies between asymptomatic and symptomatic patients with grade 3 hypertension is unclear.

**Aim:**

This study aimed to investigate cardiac and vascular HMOD in hypertensive urgencies (HU) and asymptomatic outpatients with grade 1–3 hypertension.

**Methods:**

Patients attending the ED with a symptomatic BP rise ≥180/110 mmHg were prospectively enrolled (HU group), after the exclusion of acute organ damage. HMOD and BP were assessed after 72 h from ED discharge in an office setting. These patients were matched by age and sex to outpatients with grade 3 hypertension (Grade 3 group), and by age, sex, and 72 h office BP values to outpatients with any grade hypertension (Control group).

**Results:**

A total of 304 patients were enrolled (76 patients in the HU group, 76 in the Grade 3 group, and 152 in the Control group). Grade 3 patients had increased left ventricular mass (LVMi) compared to patients with HU (106.9 ± 31.5 vs. 96.1 ± 30.7 g/m^2^, *p* = 0.035). Severe left ventricular hypertrophy (LVH) was more frequent in grade 3 (21.1 vs. 5.3%, *p* = 0.004), and pulse wave velocity (PWV) was similar in the two groups. There was no difference in LVMi between ED and Control patients (96.1 ± 30.7 vs. 95.2 ± 26.6 g/m^2^, *p* = 0.807). LVH prevalence was similar (43.4 vs. 35.5%, *p* = 0.209, respectively), but patients with HU had thicker interventricular septum (11.9 ± 2.2 vs. 11.1 ± 2.2 mm, *p* = 0.007). PWV was similar between these two groups. Patients with HU needed more antihypertensive drugs than Control patients (2 vs. 1, *p* < 0.001).

**Conclusions:**

Patients with HU had a better cardiac HMOD profile than outpatients with grade 3 hypertension. Their cardiac and vascular HMOD is more comparable to an outpatient with similar in-office BP, although they need more antihypertensive medications.

## Introduction

Arterial hypertension is an important risk factor for cardiovascular (CV) morbidity and mortality (1). Blood pressure (BP) values have an independent and continuous relationship with clinical neurovascular and CV diseases (ischemic and hemorrhagic stroke, ischemic heart disease, heart and renal failure, and peripheral artery disease) (2). Neurovascular and CV diseases are usually preceded by subclinical hypertension-mediated organ damage (HMOD), such as left ventricular hypertrophy (LVH) or arterial stiffness. The identification of subclinical HMOD is crucial for a more accurate CV risk estimation (3) and guiding clinicians in proper risk management, especially in patients with severe hypertension, in whom HMOD is common (4). Patients presenting for office evaluation with asymptomatic grade 3 hypertension had a greater prevalence of HMOD, as well as a worse CV risk profile, compared to patients with grade 1 and 2 hypertension (5). Sometimes patients with grade 3 hypertension have symptoms consistent with hypertension emergency and need urgent assessment for acute organ damage (e.g., myocardial infarction, stroke, and hypertensive encephalopathy) (6, 7). However, those in which acute organ damage is excluded, are considered as patients with symptomatic uncontrolled grade 3 hypertension, formerly known as hypertensive urgencies (8). Data on the prevalence of HMOD in patients attending the Emergency Department (ED) with symptomatic BP rise are scarce, and it is unclear whether HMOD prevalence varies between patients with asymptomatic and symptomatic grade 3 hypertension. Moreover, recent findings from an Italian survey regarding emergency and intensive care departments show how the knowledge about this topic is very heterogeneous among the medical community, both in the terms of diagnostic and therapeutic approach, and suggested follow-up after the acute phase (9).

The aim of our study was to investigate subclinical cardiac and vascular HMOD in patients with grade 1–3 arterial hypertension evaluated in the Office Setting and in patients with hypertensive urgencies referred to the ED.

## Materials and Methods

**Figure 1:**
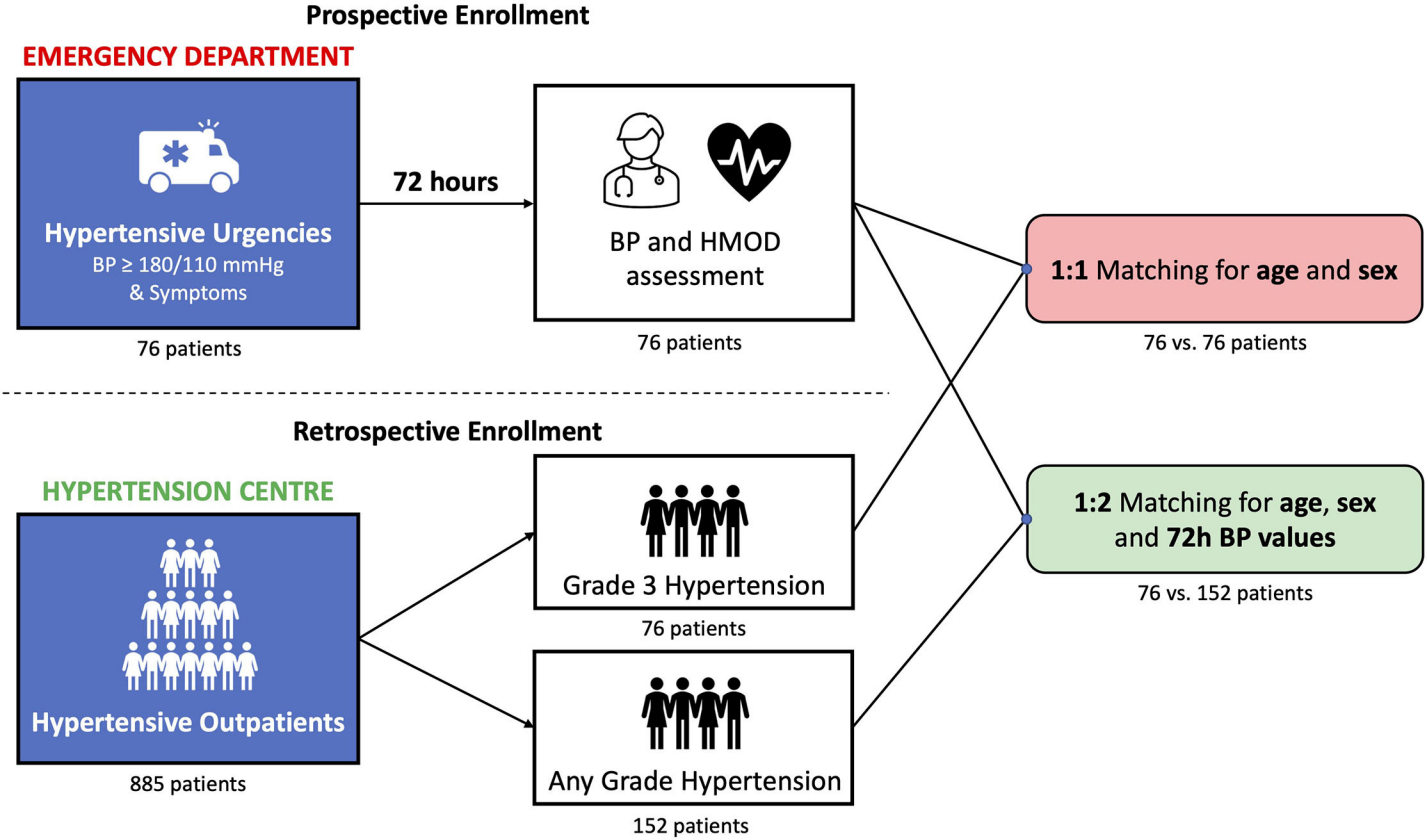
Study design. Prospective and retrospective enrollment and subsequent matching process (details in the main text). BP, blood pressure; HMOD, hypertension-mediated organ damage.

The study consisted of a subset of prospectively enrolled patients with hypertensive urgency referred to the ED and a subset of retrospectively hypertensive outpatients selected from the Office Setting (Figure 1).

### Prospective Enrollment

Patients admitted to the ED of the “Città della Salute e della Scienza” Hospital of Turin, from 1st January 2020 to 31st December 2021, were prospectively enrolled as per the following inclusion/exclusion criteria:

Inclusion criteria: symptomatic BP rise, characterized by systolic BP ≥ 180 mmHg, and/or diastolic BP (DBP) ≥110 mmHg, without acute clinical organ damage.

### Exclusion Criteria: High BP Due to Traumatic Causes or Known Neoplastic Pain, Acute Clinical Organ Damage (Hypertensive Emergency), and Denied Consent

Enrolled patients with acute BP disorders were freely managed, both diagnostically and therapeutically, by the emergency physicians in the ED, according to clinical presentations as suggested in the current European position paper (8). After exclusion of acute clinical organ damage, patients were discharged from the ED and referred for our evaluation at the Third Level Hypertension Center within 72 h of discharge. At the time of the official evaluation at the Hypertension Center, all patients were assessed for subclinical cardiac and vascular HMOD.

### Retrospective Enrolment

Patients were selected from the 8-year archive (2013–2021) of our Third Level Hypertension Center according to the following inclusion/exclusion criteria:

Inclusion criteria include a first visit to the Hypertension Center and subclinical cardiac and vascular HMOD assessment at the time of the first evaluation.

Exclusion criteria include masked or white coat hypertension, symptoms consistent with the hypertensive emergency at the time of the visit, and previous suspected or confirmed hypertensive emergency.

Meeting these criteria, hypertensive outpatients were selected through the two following matching rules. Patients with grade 3 arterial hypertension (Grade 3 group) were 1:1 matched for age and sex to prospectively enrolled patients with symptomatic. Moreover, considering that patients with HU could be present at the Hypertension Center with office BP values different from those in the ED, we performed a second 1:2 matching, by age, sex, and office BP values, to hypertensive outpatients of any grade (Control group).

### BP Measurement

In both prospectively and retrospectively enrolled patients, at any time of medical assessment, BP measurements were performed according to the current European Society of Hypertension/European Society of Cardiology (ESH/ESC) recommendations (10). Automatic sphygmomanometers were used (Omron, M10-IT models, Matsusaka, Kyoto, Japan), with study subjects in the sitting position after a 5-min rest. Three BP measurements were performed, and the mean value was used for subsequent analysis. In patients attending the ED, BP measurements were performed during the medical examination. Resistant hypertension was defined as office BP values ≥ 140/90 mmHg, despite the use of 3 or more hypertensive drugs, including a diuretic (10).

### Subclinical Cardiac HMOD—Echocardiography

Standard two-dimensional transthoracic echocardiographic images were acquired by expert European Association of Cardiovascular Imaging (EACVI) accredited staff using a commercially available ultrasound machine (IE33, Phillips Medical Systems, Andover, Massachusetts, USA) using an S5 transducer, with patients lying on their left side, following a standard echocardiographic-imaging protocol. Conventional parameters were assessed according to the current guidelines (11). A left ventricular (LV) mass was estimated. Devereux's formula was used to calculate the body surface area (BSA) and LV mass values were indexed for BSA (LVMi) and height raised to 2.7 (LVMh^2.7^) in patients with non-obese and obese, respectively (11). LV volumes, ejection fraction, and left atrial volume were assessed using Simpson's Biplane technique from apical two and four-chamber views and indexed for BSA. LV diastolic function was estimated through the evaluation of left atrial volume, mitral inflow peak systolic velocities of early (E) and late (A) diastolic filling on pulsed-wave Doppler, color-tissue Doppler imaging of the septal and lateral mitral annulus (E'), according to current international recommendations (12).

### Subclinical Vascular HMOD—Pulse Wave Velocity Analysis

Arterial stiffness was quantified using carotid-femoral pulse wave velocity (PWV). Pressure waveforms at the carotid and femoral arteries were obtained non-invasively by applanation tonometry (Sphygmocor, AtCor Medical—Sydney, Australia) (13). The distance traveled by the pressure wave was defined as the distance between the two recording sites, measured by the operator. The PWV was then calculated as the ratio between the distance traveled by the wave and the time delay between the feet of the two waveforms.

### Subclinical Organ Damage Endpoints

Alterations of LV mass and geometry increased left atrial volume, and diastolic dysfunction was considered subclinical cardiac HMOD (14). In patients with non-obese, LV hypertrophy (LVH) was defined by LVMi >115 g/m^2^ in men and >95 g/m^2^ in women. Severe LVH was defined as LVMi >122 g/m^2^ in women and >149 g/m^2^ in men (14). In patients with obese [i.e., body mass index (BMI) >30 mg/m^2^], LVH was defined by LVMh^2.7^ ≥50 g/m^2.7^ in men and ≥47 g/m^2.7^ in women (10, 11). Relative wall thickness (RWT) was defined as two-times inferolateral wall thickness divided by the LV diastolic diameter and was used to classify LV remodeling as either concentric (RWT > 0.42) or eccentric (RWT ≤ 0.42). Interventricular septum (IVS) thickness was used as a minor index of cardiac hypertensive remodeling. IVS thickness > 10 mm in men and > 9 mm in women was considered abnormal (11). Left atrial volume >34 ml/m^2^ was considered abnormal (11). PWV >10 m/s was considered as an index of abnormal aortic stiffness (10).

As recommended by current guidelines, the estimated glomerular filtration rate (eGFR) was assessed through the Chronic Kidney Disease Epidemiology Collaboration (CKD-EPI) formula, based on serum creatinine measured in the ED for patients with HU and on serum creatinine measured within 3 months of clinical evaluation for hypertensive outpatients. CKD was defined as an eGFR <60 ml/min/1.73 m^2^ (10, 15).

### Statistical Analysis

Statistical analysis was performed by dedicated software (R: A Language and Environment for Statistical Computing, v4.0.0 for Mac OSX, R Core Team., Vienna, Austria). Continuous variables were expressed as the mean ± standard deviation (SD). Qualitative variables were expressed as absolute values of frequency and percentage values. The normal distribution of variables was tested using the Kolmogorov–Smirnov and residual analysis tests. Differences between independent groups were evaluated using a *t*-test for continuous variables with normal distribution and the Mann–Whitney or Kruskal–Walli's test for continuous variables with non-normal distribution. Categorical variables were compared using the chi-square test or Fisher's exact test, as appropriate. Statistical significance was considered for the values of *p* < 0.05.

Propensity score matching was used to establish comparable patient cohorts (16). Multivariable logistic regression was used to generate the propensity score based on age and gender for ED-grade 3 matching (match 1), and on age, gender, and ambulatory systolic blood pressure (SBP) for ED-control matching (match 2). Matching was performed using a 1:1 optimal matching method protocol for match 1, and a 1:2 optimal matching method protocol for match 2. Both matching processes were performed without replacement (nearest neighbor approach) with a caliper width equal to 0.20 of the SD of the logit of the propensity score.

The present study was approved by our Institutional Review Committee (Comitato Etico Interaziendale A.O.U. Città della Salute e della Scienza di Torino—A.O. Ordine Mauriziano), and all subjects submitted their written informed consent.

## Results

A total of 103 patients attending the ED with a symptomatic BP rise were prospectively enrolled, and 76 of them met inclusion/exclusion criteria.

In total, 885 outpatients selected from the archive of our Hypertension Center met inclusion/exclusion criteria. After the matching process following the mentioned criteria, the Grade 3 group consisted of 76 patients and the Control group consisted of 152 patients.

The total study population consisted of 304 patients. There were 140 men (46%) with a mean age of 60.1 ± 13.7 years and a BMI of 27.8 ± 5.2 kg/m^2^. Median arterial hypertension duration was 10 years (IQ 3.0–19.0), and global HMOD prevalence (i.e., any of the following: LVH, LAe, and PWV > 10 m/s) was 62.5%. Other clinical and echocardiographic characteristics of the total study population and subgroups are reported in Tables 1a,b.

**Table 1a:** Demographic and clinical characteristics of total study population and subgroups.

	**Total**	**HU group**	**Grade 3 group**	**Control group**
	***N* = 304**	***N* = 76**	***N* = 76**	***N* = 152**
Male sex [*n* (%)]	140 (46.1%)	35 (46.1%)	35 (46.1%)	70 (46.1%)
Age (y)	60.1 ± 13.7	60.8 ± 13.1	59.6 ± 12.6	59.9 ± 14.6
Height (cm)	165 ± 10.4	165 ± 10.5	166 ± 10.4	165 ± 10.4
Weight (kg)	76.5 ± 17.2	80.1 ± 19.2	77.6 ± 18.0	74.0 ± 15.4
BMI (kg/m^2^)	27.8 ± 5.21	29.2 ± 5.84	28.1 ± 5.40	27.0 ± 4.61
BSA (m^2^)	1.83 ± 0.23	1.87 ± 0.25	1.85 ± 0.24	1.81 ± 0.22
ED SBP (mmHg)	–	194 ± 18.7	–	–
ED DBP (mmHg)	–	107 ± 12.9	–	–
Office SBP (mmHg)	–	144 ± 22.0	185 ± 20.1	145 ± 21.9
Office DBP (mmHg)	–	84.1 ± 15.4	101 ± 17.8	80.4 ± 12.8
Diabetes [*n* (%)]	32 (10.5%)	18 (23.7%)	6 (7.89%)	8 (5.26%)
Dyslipidemia [*n* (%)]	79 (26%)	29 (38.2%)	15 (19.7%)	35 (23.0%)
CAD [*n* (%)]	23 (7.6%)	12 (15.8%)	8 (10.5%)	3 (1.97%)
Heart failure [*n* (%)]	5 (1.6%)	4 (5.26%)	0 (0.00%)	1 (0.66%)
Atrial fibrillation [*n* (%)]	11 (3.6%)	7 (9.21%)	2 (2.63%)	2 (1.32%)
Previous stroke [*n* (%)]	15 (4.9%)	4 (5.26%)	5 (6.58%)	6 (3.95%)
CKD [*n* (%)]	32 (10.5%)	13 (17.1%)	9 (11.8%)	10 (6.6%)
Hyp duration (y) [IQ range]	10.0 [3.0; 19.0]	10.0 [4.3; 17.0]	11.0 [1.3; 18.5]	10.0 [3.0; 19.0]
Hyp drugs (*n*) [IQ range]	2.0 [1.0; 3.0]	2.0 [1.75; 3.0]	2.0 [0.0; 3.0]	1.0 [0.0; 2.0]
Hyp drugs ≥ 3 [*n* (%)]	92 (30.3%)	25 (32.9%)	30 (39.5%)	37 (24.3%)

*BMI, body mass index; BSA, body surface area; CAD, coronary artery disease; CKD, chronic kidney disease; DBP, diastolic blood pressure; ED, emergency department; Hyp, hypertension; SBP, systolic blood pressure*.

**Table 1b:** Echocardiographic characteristics of total study population and subgroups.

	**Total**	**HU group**	**Grade 3 group**	**Control group**
	***N* = 304**	***N* = 76**	***N* = 76**	***N* = 152**
LVMi (g/m^2^)	98.0 ± 29.0	96.1 ± 30.7	106.9 ± 31.7	95.2 ± 26.6
IVS (mm)	11.5 ± 2.3	11.9 ± 2.2	11.9 ± 2.55	11.1 ± 2.1
RWT	0.46 ± 0.11	0.46 ± 0.10	0.47 ± 0.12	0.46 ± 0.11
LVH [*n* (%)]	124 (40.8%)	33 (43.4%)	37 (48.7%)	54 (35.5%)
Severe LVH [*n* (%)]	35 (11.5%)	4 (5.3%)	16 (21.1%)	15 (9.9%)
EF (%)	60.3 ± 7.0	61.1 ± 8.5	60.4 ± 7.0	61.2 ± 6.2
LAVi (ml/m^2^)	28.1 ± 10.0	28.0 ± 10.3	30.6 ± 10.0	26.9 ± 9.7
Lae [*n* (%)]	76 (25.2%)	21 (27.6%)	23 (30.7%)	32 (21.2%)
SOV (mm)	34.0 ± 4.7	32.7 ± 4.1	34.0 ± 4.5	34.7 ± 5.0
ASC (mm)	35.3 ± 5.2	34.0 ± 5.0	35.9 ± 4.1	35.9 ± 5.9
E (m/s)	0.63 ± 0.18	0.70 ± 0.17	0.65 ± 0.21	0.59 ± 0.16
E/A ratio	0.93 ± 0.36	0.91 ± 0.22	0.92 ± 0.35	0.94 ± 0.45
E' lateral (cm/s)	8.21 ± 3.32	8.06 ± 2.43	7.69 ± 2.89	8.54 ± 3.85
E' septal (cm/s)	6.07 ± 2.03	6.13 ± 1.66	5.80 ± 2.00	6.18 ± 2.20
E/E' ratio	10.1 ± 4.2	10.9 ± 4.5	10.9 ± 5.1	9.2 ± 3.2
E/E' ratio > 14 [*n* (%)]	41 (13.5%)	12 (16.0%)	13 (17.6%)	16 (11.0%)
Diastolic dysfunction [*n* (%)]	140 (46.1%)	30 (39.5%)	42 (55.3%)	68 (44.7%)
TR max vel (m/s)	2.29 ± 0.33	2.33 ± 0.38	2.27 ± 0.29	2.28 ± 0.32
PWV (m/s)	9.36 ± 2.40	9.57 ± 2.18	9.89 ± 2.70	9.01 ± 2.28
PWV > 10 m/s [*n* (%)]	99 (32.6%)	24 (37.5%)	33 (43.4%)	42 (27.6%)
HMOD^*^≥ 1 [*n* (%)]	190 (62.5%)	50 (65.8%)	57 (75.0%)	83 (54.6%)

* *Any of the following: LVH, Lae, and PWV > 10 m/s*.

*E wave, transmitral inflow early wave on pulsed-wave Doppler; E/A ratio, pulsed-wave Doppler transmitral inflow E wave to transmitral inflow late (A) wave ratio; E', mitral annulus (lateral/septal) early wave on tissue-doppler imaging; E/E' ratio, mean E wave to E' wave (lateral/septal) ratio; EF, ejection fraction; HMOD, hypertension mediated organ damage; IVS, interventricular septum; Lae, left atrial enlargement; LAVi, left atrial volume indexed for body surface area; LVH, left ventricular hypertrophy; LVMi, left ventricular mass indexed for body surface area; PWV, pulse wave velocity; RWT, relative wall thickness; SOV, sinuses of Valsalva; ASC, ascending aorta; TR max vel, tricuspid regurgitation maximal velocity*.

Patients with HU had significantly higher systolic and DBP values on ED admission than on the following office evaluation, 194 ± 18.7 vs. 144 ± 22.0 mmHg (*p* < 0.001), and 107 ± 12.9 vs. 84.1 ± 15.4 mmHg (*p* < 0.001), respectively. Among patients with HU, at the time of office evaluation, 30 patients (39.5%) had BP <140/90 mmHg, 27 patients (35.5%) had grade 1 hypertension, 11 patients (14.5%) had grade 2 hypertension, and 8 patients (10.5%) had grade 3 hypertension.

Dyspnea (12 patients−15.8%), chest pain (18 patients−23.7%), headache (38 patients−50%), and neurological symptoms (17 patients−22.4%) were the most common presenting symptoms in the patients with HU. Other non-specific symptoms were described by 13 patients (17.1%).

### Comparison Between HU Group and Grade 3 Group

Clinical and echocardiographic features of the HU group and the Grade 3 group were compared (Tables 2a,b, respectively). Patients with HU had significantly higher BP values on ED admission than office BP values of patients with Grade 3 (systolic 194 ± 19 vs. 185 ± 20 mmHg, *p* = 0.004 and diastolic 107 ± 12.9 vs. 101 ± 17.8 mmHg, *p* = 0.020). Patients with Grade 3 had significantly increased LVMi than patients with HU (106.9 ± 31.5 vs. 96.1 ± 30.7 g/m^2^, *p* = 0.035). While the prevalence of LVH was similar (48.7 vs. 43.4%, *p* = 0.515) and severe LVH was more frequent in patients with Grade 3 (21.1 vs. 5.3%, *p* = 0.004). The mean LAVi was similar between patients with Grade 3 and patients with HU (30.6 ± 10.0 vs. 28.0 ± 10.3 ml/m^2^, respectively, *p* = 0.123), such as the prevalence of LAe (30.7 vs. 27.6%, respectively, *p* = 0.682). PWV values were comparable between the two groups (9.89 ± 2.70 vs. 9.57 ± 2.18 m/s in patients with Grade 3 and in patients with HU, respectively, *p* = 0.441), as well as the prevalence of pathological PWV (43.4 vs. 37.5%, *p* = 0.477). The prevalence of HMOD was 75.0% in patients with Grade 3 and 65.8% in patients with HU (*p* = 0.283). Arterial hypertension duration was similar between the two groups, as well as the median number of antihypertensive drugs (as shown in Table 2a). Resistant hypertension was present in 15 patients with HU (19.7%) and in 30 patients with Grade 3 (39.5%) (*p* = 0.008). Patients with HU had a higher prevalence of CV comorbidities, such as diabetes (*p* = 0.008), dyslipidemia (*p* = 0.012), and chronic of heart failure (*p* = 0.043). The prevalence of CKD was similar between the two groups (17.1 vs. 11.8%, *p* = 0.356). Mean eGFR was 83 ± 25 vs. 82 ± 22 ml/min/1.73 m^2^ in patients with HU and patients with Grade 3, respectively (*p* = 0.763).

**Table 2a:** Comparison between the hypertensive urgencies (HU) group and Grade 3 group (demographic and clinical characteristics).

	**HU group**	**Grade 3 group**	***p*-value**
	***N* = 76**	***N* = 76**	
Male Sex [*n* (%)]	35 (46.1%)	35 (46.1%)	1.000
Age (y)	60.8 ± 13.1	59.6 ± 12.6	0.576
Height (cm)	165 ± 10.5	166 ± 10.4	0.626
Weight (kg)	80.1 ± 19.2	77.6 ± 18.0	0.405
BMI (kg/m^2^)	29.2 ± 5.84	28.1 ± 5.40	0.207
BSA (m^2^)	1.87 ± 0.25	1.85 ± 0.24	0.656
ED SBP (mmHg)	194 ± 19^*^	–	0.004
ED DBP (mmHg)	107 ± 12.9^#^	–	0.020
Office SBP (mmHg)	–	185 ± 20	–
Office DBP (mmHg)	–	101 ± 17.8	–
Diabetes [*n* (%)]	18 (23.7%)	6 (7.89%)	0.008
Dyslipidemia [*n* (%)]	29 (38.2%)	15 (19.7%)	0.012
CAD [*n* (%)]	12 (15.8%)	8 (10.5%)	0.337
Heart failure [*n* (%)]	4 (5.26%)	0 (0.00%)	0.043
Atrial fibrillation [*n* (%)]	7 (9.21%)	2 (2.63%)	0.086
Previous stroke [*n* (%)]	4 (5.26%)	5 (6.58%)	0.731
CKD [*n* (%)]	13 (17.1%)	9 (11.8%)	0.356
Hyp duration (y) [IQ range]	10.0 [4.3;17.0]	11.0 [1.3;18.5]	0.781
Hyp drugs (*n*) [IQ range]	2.0 [1.75;3.0]	2.0 [0.0;3.0]	0.175
Hyp drugs ≥ 3 [*n* (%)]	25 (32.9%)	30 (39.5%)	0.399

* *p = 0.004 in comparison with Grade 3 Office SBP*;

*^#^p = 0.020 in comparison with Grade 3 Office DBP*.

*BMI, body mass index; BSA, body surface area; CAD, coronary artery disease; CKD, chronic kidney disease; DBP, diastolic blood pressure; ED, emergency department; Hyp, hypertension; SBP, systolic blood pressure*.

**Table 2b:** Comparison between the HU group and Grade 3 group (echocardiographic characteristics).

	**HU group**	**Grade 3 group**	***p*-value**
	***N* = 76**	***N* = 76**	
LVMi (g/m^2^)	96.1 ± 30.7	106.9 ± 31.7	0.035
IVS (mm)	11.9 ± 2.2	11.9 ± 2.55	0.952
RWT	0.46 ± 0.10	0.47 ± 0.12	0.617
LVH [*n* (%)]	33 (43.4%)	37 (48.7%)	0.515
Severe LVH [*n* (%)]	4 (5.3%)	16 (21.1%)	0.004
EF (%)	61.1 ± 8.5	60.4 ± 7.0	0.605
LAVi (ml/m^2^)	28.0 ± 10.3	30.6 ± 10.0	0.123
LAe [*n* (%)]	21 (27.6%)	23 (30.7%)	0.682
SOV (mm)	32.7 ± 4.1	34.0 ± 4.48	0.079
ASC (mm)	34.0 ± 5.0	35.9 ± 4.11	0.016
E (m/s)	0.70 ± 0.17	0.65 ± 0.21	0.093
E/A ratio	0.91 ± 0.22	0.92 ± 0.35	0.811
E' lateral (cm/s)	8.06 ± 2.43	7.69 ± 2.89	0.398
E' septal (cm/s)	6.13 ± 1.66	5.80 ± 2.00	0.281
E/E' ratio	10.9 ± 4.5	10.9 ± 5.1	0.999
E/E' ratio > 14 [*n* (%)]	12 (16.0%)	13 (17.6%)	0.798
Diastolic dysfunction [*n* (%)]	30 (39.5%)	42 (55.3%)	0.051
TR max vel (m/s)	2.33 ± 0.38	2.27 ± 0.29	0.467
PWV (m/s)	9.57 ± 2.18	9.89 ± 2.70	0.441
PWV > 10 m/s [*n* (%)]	24 (37.5%)	33 (43.4%)	0.477
HMOD^*^≥ 1 [*n* (%)]	50 (65.8%)	57 (75.0%)	0.283

* *Any of the following: LVH, Lae, and PWV > 10 m/s*.

*E wave, transmitral inflow early wave on pulsed-wave Doppler; E/A ratio, pulsed-wave Doppler transmitral inflow E wave to transmitral inflow late (A) wave ratio; E', mitral annulus (lateral/septal) early wave on tissue-doppler imaging; E/E' ratio, mean E wave to E' wave (lateral/septal) ratio; EF, ejection fraction; HMOD, hypertension mediated organ damage; IVS, interventricular septum; Lae, left atrial enlargement; LAVi, left atrial volume indexed for body surface area; LVH, left ventricular hypertrophy; LVMi, left ventricular mass indexed for body surface area; PWV, pulse wave velocity; RWT, relative wall thickness; SOV, sinuses of Valsalva; ASC, ascending aorta; TR max vel, tricuspid regurgitation maximal velocity*.

### Comparison Between HU Group and Control Group

Differences in clinical and echocardiographic characteristics between the ED and Control groups are summarized in Tables 3a,b, respectively. No significant differences were found in LVMi (96.1 ± 30.7 vs. 95.2 ± 26.6 g/m^2^, in ED and Control patients, respectively, *p* = 0.807). LVH prevalence was 43.4% in the HU group and 35.5% in the Control group (*p* = 0.209). Patients with HU had thicker IVS (11.9 ± 2.2 vs. 11.1 ± 2.2 mm, *p* = 0.007). The two groups had similar mean LAVi (28.0 ± 10.3 vs. 26.9 ± 9.7 ml/m^2^, *p* = 0.413) and LAe prevalence (27.6 vs. 21.2%, *p* = 0s.279). Patients with HU had slightly higher indexes of LV filling pressure than Control patients; in particular, E/E' ratio was 10.9 ± 4.5 and 9.2 ± 3.2, respectively (*p* < 0.001). There was no significant difference in mean PWV between the two groups (9.57 ± 2.18 vs. 9.01 ± 2.28 m/s, *p* = 0.101). The prevalence of CV HMOD was 65.8% in patients with HU and 54.6% in the Control group (*p* = 0.106). There was no difference in arterial hypertension duration (*p* = 0.992). Patients with HU assumed higher median number of antihypertensive medication than Control patients [2.0 [1.75; 3.0] vs. 1.0 [0.0; 2.0], *p* < 0.001], while the proportion of patients treated with 3 or more medications was similar (*p* = 0.171). Resistant hypertension was present in 15 patients with HU (19.7%) and in 23 Control patients (15.1%) (*p* = 0.379). The prevalence of CKD was higher in patients with HU than Control patients (17.1 vs. 6.6%, *p* = 0.013). Mean eGFR was 83 ± 25 vs. 88 ± 25 ml/min/1.73 m^2^ in patients with HU and Control patients, respectively (*p* = 0.105). Patients with HU had higher prevalence of other CV comorbidities and risk factors (Table 3a).

**Table 3a:** Comparison between the HU group and the Control group (demographic and clinical characteristics).

	**HU group**	**Control group**	***p*-value**
	***N* = 76**	***N* = 152**	
Male Sex [*n* (%)]	35 (46.1%)	70 (46.1%)	1.000
Age (y)	60.8 ± 13.1	59.9 ± 14.6	0.667
Height (cm)	165 ± 10.5	165 ± 10.4	0.868
Weight (kg)	80.1 ± 19.2	74.0 ± 15.4	0.010
BMI (kg/m^2^)	29.2 ± 5.8	27.0 ± 4.6	0.002
BSA (m^2^)	1.87 ± 0.25	1.81 ± 0.22	0.077
ED SBP (mmHg)	194 ± 19	–	–
ED DBP (mmHg)	107 ± 12.9	–	–
Office SBP (mmHg)	144 ± 22.0	145 ± 21.9	0.723
Office DBP (mmHg)	84.1 ± 15.4	80.4 ± 12.8	0.058
Diabetes [*n* (%)]	18 (23.7%)	8 (5.26%)	<0.001
Dyslipidemia [*n* (%)]	29 (38.2%)	35 (23.0%)	0.017
CAD [*n* (%)]	12 (15.8%)	3 (1.97%)	<0.001
Heart failure [*n* (%)]	4 (5.26%)	1 (0.66%)	0.025
Atrial fibrillation [*n* (%)]	7 (9.21%)	2 (1.32%)	0.004
Previous stroke [*n* (%)]	4 (5.26%)	6 (3.95%)	0.647
CKD [*n* (%)]	13 (17.1%)	10 (6.6%)	0.013
Hyp duration (y) [IQ range]	10.0 [4.3;17.0]	10.0 [3.0;19.0]	0.992
Hyp drugs (*n*) [IQ range]	2.0 [1.75;3.0]	1.0 [0.0;2.0]	<0.001
Hyp drugs ≥ 3 [*n* (%)]	25 (32.9%)	37 (24.3%)	0.171

*BMI, body mass index; BSA, body surface area; CAD, coronary artery disease; CKD, chronic kidney disease; DBP, diastolic blood pressure; ED, emergency department; Hyp, hypertension; SBP, systolic blood pressure*.

**Table 3b:** Comparison between the HU group and the Control group (echocardiographic characteristics).

	**HU group**	**Control group**	***p*-value**
	***N* = 76**	***N* = 152**	
LVMi (g/m^2^)	96.1 ± 30.7	95.2 ± 26.6	0.807
IVS (mm)	11.9 ± 2.18	11.1 ± 2.10	**0.007**
RWT	0.46 ± 0.10	0.46 ± 0.11	0.751
LVH [*n* (%)]	33 (43.4%)	54 (35.5%)	0.209
Severe LVH [*n* (%)]	4 (5.3%)	15 (9.9%)	0.236
EF (%)	61.1 ± 8.5	61.2 ± 6.2	0.862
LAVi (ml/m^2^)	28.0 ± 10.3	26.9 ± 9.7	0.413
LAe [*n* (%)]	21 (27.6%)	32 (21.2%)	0.279
SOV (mm)	32.7 ± 4.1	34.7 ± 5.0	**0.003**
ASC (mm)	34.0 ± 5.0	35.9 ± 5.9	**0.025**
E (m/s)	0.70 ± 0.17	0.59 ± 0.16	**<0.001**
E/A ratio	0.91 ± 0.22	0.94 ± 0.45	0.612
E' lateral (cm/s)	8.06 ± 2.43	8.54 ± 3.85	0.325
E' septal (cm/s)	6.13 ± 1.66	6.18 ± 2.20	0.879
E/E' ratio	10.9 ± 4.5	9.2 ± 3.2	**<0.001**
E/E' ratio > 14 [*n* (%)]	12 (16.0%)	16 (11.0%)	0.295
Diastolic dysfunction [*n* (%)]	30 (39.5%)	68 (44.7%)	0.449
TR max vel (m/s)	2.33 ± 0.38	2.28 ± 0.32	0.459
PWV (m/s)	9.57 ± 2.18	9.01 ± 2.28	0.101
PWV > 10 m/s [*n* (%)]	24 (31.6%)	42 (27.6%)	0.151
HMOD^*^≥ 1 [*n* (%)]	50 (65.8%)	83 (54.6%)	0.106

* *Any of the following: LVH, Lae, and PWV > 10 m/s*.

*E wave, transmitral inflow early wave on pulsed-wave Doppler; E/A ratio, pulsed-wave Doppler transmitral inflow E wave to transmitral inflow late (A) wave ratio; E', mitral annulus (lateral/septal) early wave on tissue-doppler imaging; E/E' ratio, mean E wave to E' wave (lateral/septal) ratio; EF, ejection fraction; HMOD, hypertension mediated organ damage; IVS, interventricular septum; Lae, left atrial enlargement; LAVi, left atrial volume indexed for body surface area; LVH, left ventricular hypertrophy; LVMi, left ventricular mass indexed for body surface area; PWV, pulse wave velocity; RWT, relative wall thickness; SOV, sinuses of Valsalva; ASC, ascending aorta; TR max vel, tricuspid regurgitation maximal velocity*.

## Discussion

In the present study, we analyzed subclinical cardiac and vascular HMOD indexes in patients admitted to the ED with an acute BP rise (i.e., BP ≥ 180/110 mmHg and consistent symptoms) in which acute organ damage has been excluded, to better understand the CV risk profile of this often-neglected specific population. We compared these patients with hypertensive outpatients with similar BP values, considering both BP values on ED admission (matching to Grade 3 outpatients) and the subsequent office BP values after 72 h of ED discharge (matching to Control patients).

To the best of our knowledge, this is the first study comparing patients with symptomatic BP rise without acute organ damage (hypertensive urgencies) to ambulatory outpatients through this specific matching.

We found that patients with HU had minor subclinical cardiac HMOD compared with Grade 3 outpatients. They had similar prevalence of LVH (43.4 vs. 48.7%, *p* = 0515), but a lower prevalence of severe LVH (5.3 vs. 21.1%, *p* = 0.004) and lower LVMi (96.1 ± 30.7 vs. 106.9 ± 31.7 g/m^2^, *p* = 0.035), despite the significantly worst CV risk profile, in terms of comorbidities. Subclinical vascular HMOD was comparable between the two groups, in terms of PWV (9.57 ± 2.18 vs. 9.89 ± 2.70 m/s, *p* = 0.441) and pathological arterial stiffness (37.5 vs. 43.4%, *p* = 0.477). On the other hand, patients with HU had similar HMOD features compared with Control patients. Only minor indices of subclinical cardiac HMOD, such as mean IVS thickness and E/E' ratio, resulted to be significantly worse in patients with HU.

Our data are consistent with those described in a large meta-analysis, where echocardiographic LVH was present in 36–41% of patients with hypertension (17). Data on the prevalence of arterial stiffness assessed with the PWV method are scarce, mainly because of different cut-offs available according to age and BP (18). We decided to use the cut-off value of 10 m/s as suggested by current guidelines on the management of arterial hypertension (10).

These results suggest that patients with HU have HMOD features comparable with patients presenting with similar BP values in an ambulatory setting without acute symptoms. Grade 3 outpatients have BP values ≥ 180/110 mmHg in an ambulatory setting and are probably more prone to have such values persistently during their daily life. On the contrary, patients with HU probably experience an acute BP rise over their average BP values which are much lower. In accordance with this hypothesis, the prevalence of resistant hypertension was higher in Grade 3 than in patients with HU. A more severe cardiac organ damage found in patients with Grade 3, in terms of both LVMi and the prevalence of severe LVH, is consistent with this aspect, as it is known that LVM increases with BP values and poor BP control (17, 19). This finding is even more relevant if we consider that patients with HU have more comorbidities than patients with Grade 3.

The recent European position paper (8) proposed to abandon the term “hypertensive urgency,” because the CV risk of these patients seems not particularly high, and the rate of short-term major CV events is very low (20, 21). Moreover, grade 3 hypertensive outpatients showed a similar incidence of CV events at 6 months (0.9%) compared with hospitalized and discharged propensity-matched HU (22). Hence the Task Force indication to avoid intravenous antihypertensive treatment and manage these patients by modifying the current oral therapy, as it should be done for outpatients (8). Our findings on chronic organ damage, although lacking follow-up and thus major CV outcomes, seem to indirectly support this evidence.

On the other hand, even if patients with HU seem similar to outpatients with analogous office BP values (Control group), in terms of LVH and increased arterial stiffness prevalence, some indices of cardiac HMOD, such as mean IVS thickness and E/E' ratio, are more impaired. The role of septal hypertrophy in patients with hypertension is emerging as a marker of hypertension-induced LV remodeling and impairment (23). Additionally, patients with HU took a higher median number of hypertensive medications compared to Control patients, indicating a higher need for hypertension treatment and a harder time controlling hypertension. These data are not sufficient to define these patients as an intermediate risk category, both for the study design and the interfering comorbidities influencing LVH (24). However, it might be a clue in favor of this hypothesis that patients with occasional acute BP rises had an increase in stroke long-term risk (25) and fatal or non-fatal CV events (26) compared to controls, despite similar BP levels during follow-up. This hypothesis needs to be confirmed with future longitudinal studies.

## Limitations

Our study has some limitations that need to be addressed. First, this is an observational transversal study that has no follow-up data, and thus, we cannot express a prognostic judgment on these patients at the moment. However, we provided data on cardiac and vascular HMOD that are strong indicators of CV risk and prognosis in the hypertensive population. Second, patients with HU were prospectively enrolled, while ambulatory patients (Grade 3 and Control groups) were retrospectively enrolled. However, the matching process should partially compensate for this issue. Third, the retrospectively enrolled outpatients came from a Third Level Hypertension Center and therefore subject to possible selection bias. Fourth, the three subpopulations included in the study are different in terms of CV disease (e.g., coronary artery disease, chronic kidney disease, and diabetes). This could make some results unclear. However, some of them are strengthened, as we have previously addressed in the discussion section. Finally, the prevalence of resistant hypertension was estimated from office BP data, without the possibility to confirm inadequate BP control by home or ambulatory BP monitoring, and without data on therapeutic adherence.

## Conclusion

Patients with HU constitute a peculiar subtype of the hypertensive population. They had a better cardiac HMOD profile than patients with sustained asymptomatic grade 3 hypertension. They had similar HMOD features compared with outpatients matched for BP values in an ambulatory setting, but higher needs for antihypertensive drugs and worst minor indices of subclinical cardiac HMOD. Thus, patients with HU might have more difficult-to-control hypertension and an intermediate CV risk between grade 3, on one hand, and grade 1–2 hypertension, on the other hand.

## Data Availability Statement

The raw data supporting the conclusions of this article will be made available by the authors, without undue reservation.

## Ethics Statement

The studies involving human participants were reviewed and approved by Comitato Etico Interaziendale A.O.U. Città della Salute e della Scienza di Torino or A.O. Ordine Mauriziano. The patients/participants provided their written informed consent to participate in this study.

## Author Contributions

(1) Research project—FVa, MC, and AM: conception. FVa, AM, FVe, and EL: organization. FVa, MC, LM, AA, GM, MT, and DL: execution. (2) Statistical analysis—FVa, MC, and LA: design. MC and LA: execution. (3) Manuscript—FVa and MC: writing of the first draft. All authors: review and critique. All authors contributed to the article and approved the submitted version.

## Conflict of Interest

The authors declare that the research was conducted in the absence of any commercial or financial relationships that could be construed as a potential conflict of interest.

## Publisher's Note

All claims expressed in this article are solely those of the authors and do not necessarily represent those of their affiliated organizations, or those of the publisher, the editors and the reviewers. Any product that may be evaluated in this article, or claim that may be made by its manufacturer, is not guaranteed or endorsed by the publisher.

## Abbreviations

BMI: body mass index
BP: blood pressure
BSA: body surface area
CAD: coronary artery disease
CKD: chronic kidney disease
CV: cardiovascular
E wave: transmitral inflow early wave on pulsed-wave Doppler
E/A ratio: pulsed-wave Doppler transmitral inflow E wave to transmitral inflow late (A) wave ratio
E': mitral annulus (lateral/septal) early wave on tissue-Doppler imaging
E/E&#8217: ratio, mean E wave to E&#8217 wave (lateral/septal) ratio
EF: ejection fraction
HMOD: hypertension mediated organ damage
IVS: interventricular septum
LAe: left atrial enlargement
LAVi: left atrial volume indexed for body surface area
LV: left ventricle
LVH: left ventricular hypertrophy
LVMi: left ventricular mass indexed for body surface area
PWV: pulse wave velocity
RWT: relative wall thickness
SOV: sinuses of Valsalva
TR max vel: tricuspid regurgitation maximal velocity.
